# Design and Evaluation of a Surface Electromyography-Controlled Steering Assistance Interface

**DOI:** 10.3390/s19061308

**Published:** 2019-03-15

**Authors:** Edric John Cruz Nacpil, Zheng Wang, Rencheng Zheng, Tsutomu Kaizuka, Kimihiko Nakano

**Affiliations:** 1 Institute of Industrial Science, The University of Tokyo, 4-6-1 Komaba, Meguro-ku, Tokyo 153-8505, Japan; z-wang@iis.u-tokyo.ac.jp (Z.W.); tkaizuka@iis.u-tokyo.ac.jp (T.K.); knakano@iis.u-tokyo.ac.jp (K.N.); 2 School of Automotive Engineering, Dalian University of Technology, No. 2 Linggong Road, Ganjingzi District, Dalian 116024, China; topzrc@dlut.edu.cn

**Keywords:** human-machine interface (HMI), surface electromyography (sEMG), advanced driver assistance system (ADAS), automated driving

## Abstract

Millions of drivers could experience shoulder muscle overload when rapidly rotating steering wheels and reduced steering ability at increased steering wheel angles. In order to address these issues for drivers with disability, surface electromyography (sEMG) sensors measuring biceps brachii muscle activity were incorporated into a steering assistance system for remote steering wheel rotation. The path-following accuracy of the sEMG interface with respect to a game steering wheel was evaluated through driving simulator trials. Human participants executed U-turns with differing radii of curvature. For a radius of curvature equal to the minimum vehicle turning radius of 3.6 m, the sEMG interface had significantly greater accuracy than the game steering wheel, with intertrial median lateral errors of 0.5 m and 1.2 m, respectively. For a U-turn with a radius of 7.2 m, the sEMG interface and game steering wheel were comparable in accuracy, with respective intertrial median lateral errors of 1.6 m and 1.4 m. The findings of this study could be utilized to realize accurate sEMG-controlled automobile steering for persons with disability.

## 1. Introduction

On confined residential roads or parking areas where sharp turns are rapidly executed at or below the speed limit of 30 km/h, a primary task for automobile drivers is to be safe by keeping their “eyes on the road and hands on the steering wheel” [1,2,3]. However, using one or both hands to rotate the steering wheel rightward from 0° to 65° with an average time of 0.268 and a standard deviation (SD) of 0.065 s, results in dangerously high shoulder joint forces that could overload the supraspinatus shoulder muscle [4,5,6]. Although the risk of muscle overload is applicable to a large population of 38 million regular drivers in the United Kingdom, drivers continue to operate steering wheels frequently in modern production automobiles [4]. A further issue with some steering wheels is an increasing amount of torque input from the driver as the maximum steering wheel angle (SWA) is approached. This reduction in steering ability, i.e., reduced steering portability, mainly results from reaction forces between tires and the road and is most pronounced when an automobile is at a full stop [7,8].

**Figure 1:**
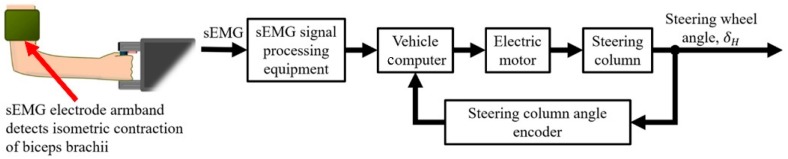
Overall steering assistance control design.

Some steering wheel users who may experience shoulder muscle overload or reduced steering portability are restricted to one-handed steering wheel operation due to hemiplegia, i.e., paralysis on one side of the body, or an amputated upper limb [9,10,11,12]. In order to reduce the risk of shoulder injury to these drivers and to avoid reduced steering portability, a steering assistance interface was developed to enable remote steering wheel rotation with only one hand. The interface included a handle to stabilize the unaffected arm of the driver and a set of surface electromyography (sEMG) electrodes positioned on the upper arm by an armband (Figure 1). Thus, the steering assistance interface will sometimes be referred to henceforth as the “sEMG interface”.

In an actual automobile, an onboard vehicle computer would rely on the sEMG electrodes to detect electrical muscle activity, i.e., myoelectric activity, resulting from isometric contractions of the biceps brachii (Figure 1) [13]. The computer converts the myoelectric activity into control signals for an electric motor. A steering column is rotated by the motor at a constant steering wheel rate (SWR) to change the SWA. 

Although sEMG electrodes have been used to reliably control prosthetic limbs and to objectively evaluate the muscle burden, steering comfort, and the time for drivers to apply force on steering wheels during vehicle turning maneuvers, few studies have investigated sEMG controlled steering as a means of assisting drivers with disability [14,15,16,17,18,19,20,21,22,23,24]. In contrast, other sensors such as joysticks, strain gauges and motion detectors have been developed [25,26,27,28,29]. Past sensor interfaces have often required force or motion input from limbs, as in the case of joysticks and gyros. However, some health conditions such as upper limb amputation and hemiplegia inhibit or preclude this input. In contrast, sEMG electrodes are more versatile because they can detect muscle signals from amputated limbs and a variety of other body parts [30]. The proposed sEMG interface could thus be readily adjusted to accommodate sEMG input from amputated limbs, in addition to intact limbs [15,16,17,30,31,32]. 

Since the safety of the test subjects was of the highest priority during the study, the proposed steering assistance interface was tested with a driving simulator as a safer alternative to an actual automobile. A further advantage over an actual automobile was the ability to use the driving simulator to execute turning maneuvers more consistently for each test subject. Myoelectric activity was measured by individual self-adhesive electrodes to provide steering control signals. If the mounting configuration of the electrodes was at least experimentally comparable to a game steering wheel, with respect to path-following accuracy, the configuration would be adapted to an automobile as an electrode armband for more efficient mounting (Figure 1).

Since path following is the primary task of conventional automobile steering, the sEMG interface was subjected to driving simulator trials to evaluate path-following accuracy in comparison to a game steering wheel [33]. Drivers used the game steering wheel and the sEMG interface to perform rightward U-turns with differing radii of curvature. U-turns allow the simulated automobile to reach steady-state cornering conditions under which SWA, turning radius, and vehicle speed are constant [6]. Therefore, U-turns were chosen as driving scenarios to clearly observe any effects from the steady-state phenomena, understeer or oversteer, that reduce path following accuracy [34]. 

One driving scenario involved a right U-turn with a radius of 3.6 m, which was equal to the minimum turning radius of the simulated automobile. The trajectory of this U-turn required the SWA to change from 0° to 65° to evaluate the accuracy of the sEMG interface in a driving scenario associated with high shoulder joint forces in steering wheel users [4]. Since the trajectory of this U-turn corresponded to the maximum SWA, it was not possible to perform understeer correction through rightward steering. The radius of the other right U-turn was two times longer at 7.2 m with a smaller corresponding SWA that allowed for understeer correction. U-turn trajectory data generated by human participants confirmed the hypothesis that, for a radius of 3.6 m, the sEMG interface would be comparable or greater in path-following accuracy to the game steering wheel. It was also confirmed, for a radius of 7.2 m, that the accuracy of the sEMG interface was at least comparable to the game steering wheel interface, even if no steering wheel correction was used with the sEMG interface. 

The rest of this paper is structured as follows: Section 2 describes the design of the sEMG interface, the selection of steering input sensors for the interface, and the adaptation of the interface to a driving simulator. The experimental setup and methodology for evaluating the path-following accuracy of the interface are also described. Section 3 conveys experimental results, whereas interpretation of the results is covered in Section 4. Finally, Section 5 provides conclusions about the experiment, including implications for future studies. 

## 2. Materials and Methods

As described in Section 2.1, the steering assistance interface integrates sEMG sensors into an automobile. The justification for the selection of sEMG electrodes over other steering input sensors is provided in Section 2.2. In order to evaluate the path-following accuracy associated with sEMG electrodes, driving simulator trials were conducted. Consequently, Section 2.2 describes how interface features relevant to sEMG measurement were adapted to a driving simulator. The overall experimental setup for driving simulator trials is detailed in Section 2.3, whereas the scenarios that were completed with the setup are described in Section 2.4. The lateral vehicle dynamics involved in the scenarios are explained in Section 2.5, whereas the procedure for evaluating path-following accuracy in the context of the scenarios is provided in Section 2.6.

### 2.1. Steering Assistance Interface

**Figure 2:**
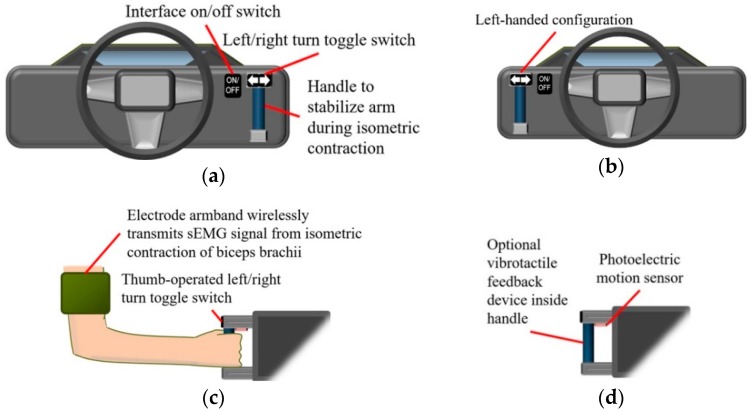
Steering assistance interface for (**a**) right-handed operation and (**b**) left-handed operation. (**c**) Steering direction selected with toggle switch before steering is initiated by surface electromyography (sEMG) signals from isometric contraction of biceps brachii. (**d**) Holding handle causes photoelectric motion sensor to activate reception of sEMG signals by steering assistance system, whereas releasing handle deactivates signal reception.

The steering assistance interface (Figure 2) enables remote steering wheel rotation to prevent decreased steering portability as the SWA increases, while reducing the risk of shoulder injury during rapid steering [3]. Unlike conventional steering wheels, the interface only needs to be operated with one hand. Thus, drivers with disabilities, such as amputees with at least one fully functioning arm, could readily operate the interface. Input from the driver is provided through sub-second sEMG signal pulses resulting from the isometric contraction of the biceps brachii [35]. A handle that is gripped to stabilize the arm during isometric contraction could be installed on the dash or at another convenient cabin location to allow left-handed or right-handed operation. 

In order to prevent steering caused by inadvertent myoelectric activity or sEMG signal noise, the handle could be released to cause a photoelectric sensor on the handle to deactivate sEMG signal reception [36]. Alternatively, the on/off toggle switch could be pressed to shut down the steering assistance system. The on/off toggle switch is also used to turn off steering assistance so that the driver can resume manual control of the steering wheel. The driver could also manually rotate the steering wheel so that a torque sensor located in the steering wheel could turn off steering assistance. 

**Figure 3:**
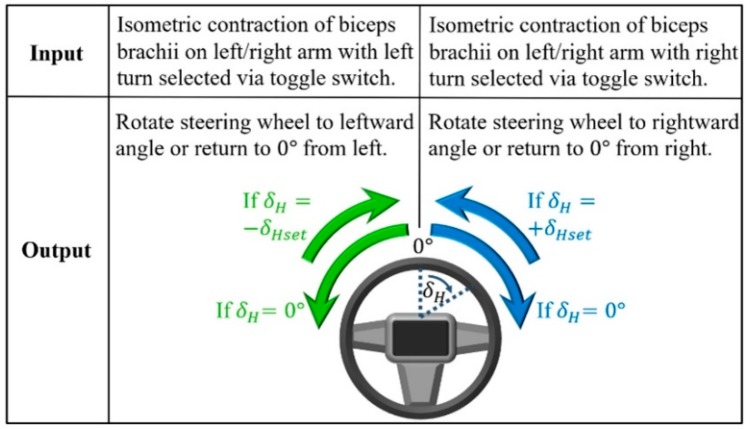
Relation between muscle contraction input and steering wheel angle output.

Turning maneuvers are executed through a finite state machine (FSM) control scheme that divides the SWA of the steering wheel into three states (Figure 3) [18]: (1) rightward SWA, +δ_Hset_, (2) leftward SWA, −δ_Hset_, and (3) the neutral position, where δ_H_ is 0°. “Set” in this case refers to the rightward and leftward SWAs being programmed into the vehicle computer according to the health conditions of drivers (Figure 1). If drivers require assistance with turns involving maximum steering wheel rotation, then maximum SWA values for +δ_Hset_ and −δ_Hset_ are programmed. Assistance with partial steering wheel rotation is provided by setting lower SWA values. One advantage resulting from lower SWA values is the ability of drivers to correct for understeer by resuming manual control of the steering wheel before steering further in the direction of the turn. 

When a rightward turning direction is selected with the left/right turn toggle switch (Figure 2c) and the biceps brachii of the preferred arm undergoes a sub-second isometric contraction, i.e., the arm is static as the biceps brachii maintains a constant length during a contraction lasting less than one second, the steering wheel is rotated rightward, i.e., clockwise from the perspective of the driver, if the initial SWA is equal to the neutral position of 0° (Figure 3) [37]. If the steering wheel is already rotated rightward to the set SWA, the same muscle contraction returns the steering wheel to the neutral position. Selecting the leftward turning direction, on the other hand, causes the steering wheel to rotate towards the left instead of the right. Regardless of the direction of rotation, the steering wheel rotates at a constant SWR. The SWR depends on the chosen steering actuator with commercially available specifications ranging from 720 to 1300 deg/s [38]. Since some steering actuators produce more steering wheel torque than human drivers, increased steering wheel torque required at low vehicle speeds could be provided by the actuators [7]. 

The driver is made aware of the SWA throughout a turn by receiving visual feedback from the position of the vehicle in relation to roadside curbs, painted road lines, and other surrounding objects. Although visual feedback is sufficient for turning, supplementary feedback could be obtained by observing the physical rotation of the steering wheel or through a vibrotactile feedback device inside the handle of the interface (Figure 2d). An existing example of a vibrotactile feedback device preferred by some prosthetic hand users, as a opposed to a lack of vibrotactile feedback, has a mass of less than 1 kg that is spun by a small direct current (dc) motor to provide a mechanical vibration to the user between 10 to 500 Hz [39,40]. Given the preference of this device among some prosthesis users, the device could be employed by the sEMG interface so that a vibration could occur for a brief period, e.g., 1 s, when the SWA completely transitions to any of the three control scheme states (Figure 3). 

**Figure 4:**
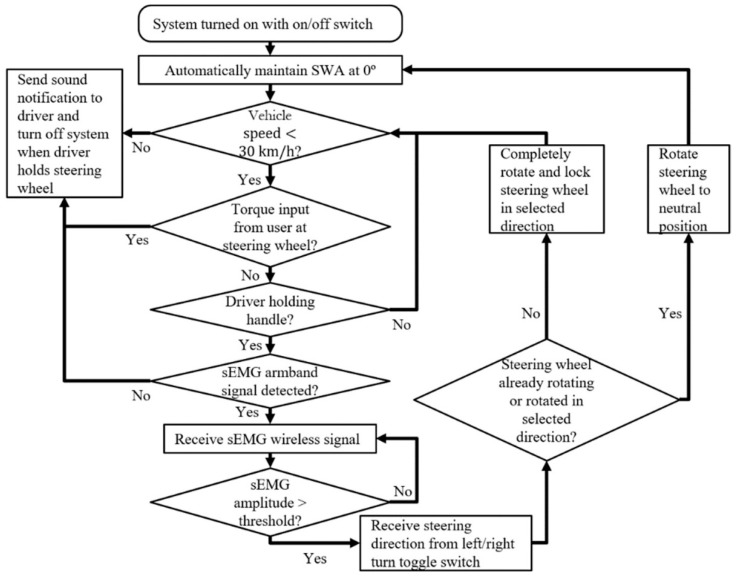
Operation flowchart of steering assistance interface.

The operation of the steering assistance interface in an actual automobile is shown as a diagram in Figure 4. After confirming that the steering wheel is at or near the neutral position, the driver turns on the steering assistance system so that the vehicle computer maintains the SWA at 0°. Since the interface is intended for steering on residential roads in Japan, where the legally prescribed speed limit for safely driving on residential roads is 30 km/h, steering assistance is automatically turned off if the vehicle speed exceeds 30 km/h [3]. The cutoff speed may be adjusted on the basis of empirical tests to determine the highest speed at which a vehicle can stably turn at the set SWR [34]. When steering assistance is turned off due to the exceeded cutoff speed or the lack of a wireless signal from the electrode armband, a sound notification such as a constant 1 s tone would alert the driver to resume manual control of the steering wheel.

According to Figure 4, if the vehicle is traveling below the cutoff speed and no input from the driver is detected at the steering wheel by a torque sensor similar to those found on automated vehicles, the interface handle could be grasped so that a photoelectric motion sensor would detect the hand of the driver and activate sEMG signal reception (Figure 2d) [41]. Before providing muscle contraction input, the steering direction is selected with a toggle switch (Figure 2c). If the sEMG signal resulting from the isometric contraction of the biceps brachii is greater than a preset threshold, e.g., 30% of the maximum sEMG amplitude, then the steering column of the vehicle is rotated in accordance with the control scheme shown in Figure 3. 

The steering assistance operation shown in Figure 4 is an instance of Level 1 to Level 3 automation, as defined by SAE (Society of Automotive Engineers) International [42]. In contrast to higher levels of automation that assign control of acceleration, deceleration, and steering to the vehicle, the user of the steering assistance interface controls acceleration and deceleration with the option to share steering control with the vehicle. Although higher levels of automation would relieve persons with disability from the task of driving, there are a number of challenges for highly automated vehicles, including: sampling mismatch among different automated driving sensors, high manufacturing cost, susceptibility of networked automation computers to hackers, high vehicle power consumption by automation hardware, and the reduced ability of advanced driver assistance systems (ADASs) to detect road obstacles under rainy or extreme lighting conditions [27,43,44,45]. Since the sEMG interface is at Level 3 or lower, such challenges could be avoided by implementing the sEMG interface over other more highly automated control schemes.

### 2.2. Selection and Configuration of Surface Electromyography (sEMG) Equipment

Various sensors have been developed for controlling automotive steering systems. Joysticks have facilitated the rotation of steering wheels, and in some instances, joysticks were designed to increase safety by replacing steering wheels that may collide with the driver in a frontal collision [28,29,46,47]. Some steer-by-wire joysticks employed electrical sensors connected to computers to perform turning maneuvers without a steering column. However, regulations applicable to countries such as Sweden and Japan prohibited the unauthorized modification of production vehicles with alternative steering controls, including steer-by-wire devices [28,48]. In order to install a joystick for drivers with disabilities, it was possible to request permission from the government of Japan, provided that the electrical sensors of the joystick controlled the steering column with an electric motor and the steering wheel remained usable [28]. Since the proposed sEMG interface was developed in Japan, the electrodes of the interface were designed to control an electric motor that rotates the steering column (Figure 1). 

Aside from sEMG electrodes, other sensors have been proposed to control steering with the bodily movements of the driver. Strain gauges, motion sensors, rotary encoders and gyros have converted gestures into steering commands [25,26,27]. Motion sensors had limited mounting locations in the vehicle cabin, with some consumer grade sensors positioned at least 50 cm from a detectable gesture [26]. Gyros that were mounted on the limbs of drivers, on the other hand, could be used in the confines of a vehicle cabin, but gyro signals were subject to drift, in addition to interference resulting from vehicle vibrations [25,26,49]. Strain gauges mounted on the hands of drivers were unaffected by vehicle vibrations or drift when detecting hand gestures [25]. Thorough testing has not been performed, however, apart from driving an automobile along a straight path. 

There is a challenge that is common to some existing steering sensor configurations, namely, the accommodation of drivers with disabilities. If amputation or other health conditions preclude the use of some limbs, strain gauges and rotary encoders require mechanical adapters to measure the movement of remaining unaffected limbs [25,27]. Likewise, joysticks designed for hands may need to be adapted to the prosthetic terminal devices of amputees [50,51]. However, the use of prosthetics is not generally accepted by amputees, since only 30% of Korean amputees were satisfied with prostheses, and 56% of amputees in Australia wear prosthetics “once in a while” or “never” [11,12]. On the other hand, electroencephalography (EEG) sensors allow brain signals to control steering without limb movement, but empirical studies revealed that about 20% of users are unable to sufficiently operate brain control interfaces [52,53,54,55]. Steering control without limb movement may be enabled by eye gaze tracking, but the “Midas touch problem” has to be addressed so that algorithms can distinguish gazes that convey commands from gazes that merely obtain visual information [56,57]. The current sEMG interface avoids this problem by accepting myoelectric signals rather than eye gaze input. 

Since sEMG electrodes could be individually mounted in selected locations, the proposed steering assistance interface could be readily adjusted to measure residual muscles from amputated limbs as in the case of sEMG-controlled prostheses [30,58]. If an amputee prefers to grip the handle with an unaffected limb, it would be possible to measure sEMG input signals from the affected limb, provided that the myoelectric activity of residual muscles could be detected with electrodes [59]. The interface could also be used by drivers, such as patients with stroke-induced hemiplegia, with one paralyzed arm and one unaffected arm that provides sEMG signals [9,10]. Even though spinner knobs could be mounted to steering wheels to enable one-handed operation in place of the proposed interface, some drivers with disabilities choose not to use spinner knobs [60]. Furthermore, it seems that further research needs to be conducted to determine whether or not the use of spinner knobs poses a risk of shoulder muscle injury, as observed when steering wheels are directly rotated with one hand [4]. 

Interfaces with sEMG sensors have their own set of challenges. Inaccurate measurement could result from motion artifacts due to the movement of electrode wires and relative motion between electrodes and skin surfaces [61]. Inaccuracy could also result from electromagnetic interference originating from body tissue and environmental sources, such as power lines and electronic devices [61]. However, signal filtering, bipolar electrode configurations, and non-polarized electrodes could mitigate electromagnetic interference [30,61]. Wireless sEMG signal transmission could also reduce noise and prevent driver movements from being impeded by electrode wires [62,63]. 

**Figure 5:**
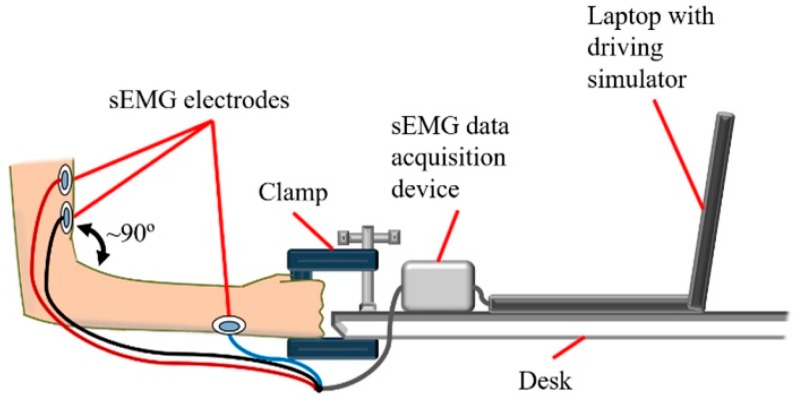
Steering assistance interface adapted to driving simulator.

Recently developed sEMG sensors for research and commercial applications utilize wireless transmission [64,65]. Unlike conventional wet electrodes that require conductive gel at the skin–electrode interface, emerging sensor technology can detect myoelectric activity without gel, and in the case of capacitive electrodes, without skin contact [64,66,67]. However, unlike some emerging sEMG sensors, disposable silver–silver chloride (Ag/AgCl) wet electrodes are relatively affordable and readily available [67]. Thus, wet electrodes were used in the current study to facilitate experimental replicability and the iteration of electrode mounting configurations (Figure 5) [30]. If the mounting configuration of the conventional electrodes corresponded to path following accuracy that was comparable to a game steering wheel, the configuration would be finalized for conversion to an armband that uses dry electrodes with comparable measurement accuracy [67]. 

Electrode placement for the driving simulator trials was performed in accordance with the recommendations of SENIAM (Surface EMG for the Non-Invasive Assessment of Muscles) [68]. The ground electrode was mounted on the wrist (Figure 5), while a set of two bipolar electrodes was placed on the belly of the biceps brachii with the elbow flexed to approximately 90°, since prior testing has shown that the muscle belly is more active at 90° of flexion than other mounting locations on the biceps brachii [69]. As the biceps brachii contracts isometrically, the hand grips a clamp that substitutes for the original handle depicted in Figure 2. Controls from the original interface design, such as a left/right turn toggle switch and photoelectric sensor, were not incorporated into the clamp, since simulator trials only included right turns that required the clamp to be held continuously (Section 2.4); isometric contractions only initiated and terminated right turns. 

Relocating the ground electrode from the wrist to a location above the elbow, such as the lateral side of the upper arm, could accommodate some amputees [70]. Whereas the buttons of the interface could be pushed with an intact arm or prosthetic limb, transradial amputees with residual muscles above the wrist could choose to provide steering input with sEMG signals from the residual biceps brachii, although residual forearm muscles that commonly control prosthetic hands may be more preferable to the user [58]. On the other hand, transhumeral amputees could typically provide sEMG signals with residual muscles above the elbow, including biceps brachii, that are capable of isometric contraction [71,72]. Both types of amputations account for a significant portion of amputees that could control the interface with affected limbs [11,12].

Similar to the majority of studies on sEMG-controlled prosthetics, nondisabled drivers in the current study performed isometric contractions that could represent sEMG signals from the affected limbs of transhumeral amputees [73,74,75,76,77]. Consequently, the results of this study are relevant to the operation of the sEMG interface with intact or affected limbs. Regardless of the presence or absence of health conditions, anatomical and physiological differences across drivers could affect the measurement of sEMG signals [61]. In order to detect sEMG amplitudes that vary across a range of drivers, the sEMG input threshold of the steering assistance interface would have to be calibrated for each driver (Section 2.1) [78,79,80]. Hence, the calibration process for driving simulator trials is described in the next section. 

### 2.3. Experimental Setup

**Figure 6:**
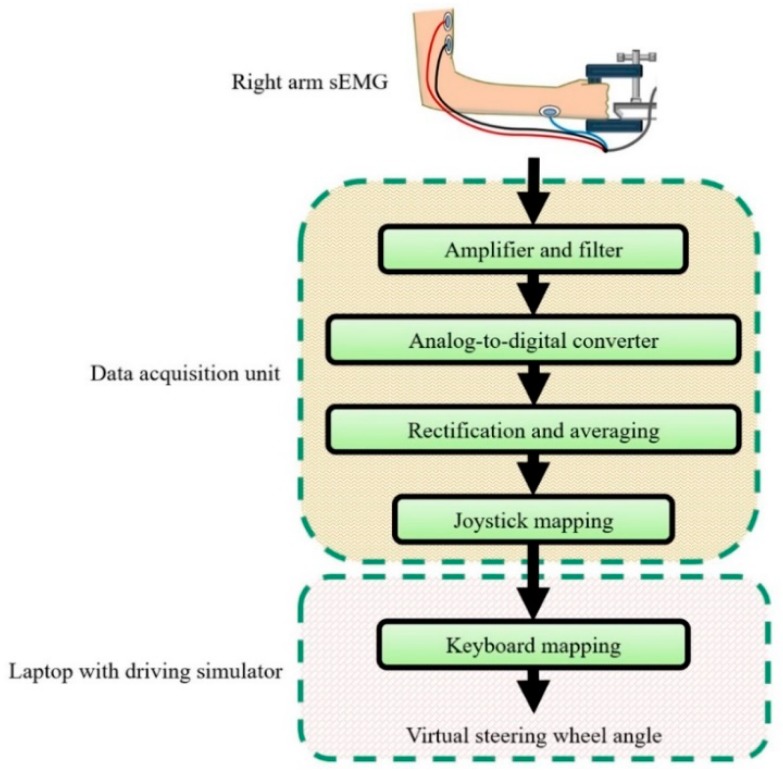
Steering assistance control system for driving simulator.

The equipment for controlling the driving simulator with the steering assistance interface is diagramed in Figure 6. Rather than controlling a physical steering wheel, sEMG signals control the virtual SWA of the driving simulator in order to facilitate the implementation of the interface. Consequently, in contrast to the original operation of the sEMG interface (Figure 4), the steering wheel torque sensor, sound notification, and other features associated with manual takeover of the steering wheel were not included in the control system. 

The sEMG data acquisition device (DAQ) consisted of a custom circuit that filtered signals below 2 Hz and above 530 Hz and amplified the remaining signals (Figure 6). The amplification stage of the circuit introduced a gain of 50 to the sEMG signal, while the subsequent rectification stage applied a gain of 100. Hence, the combined gain, which is equal to the product of the gains from each stage, was equal to 5000 (Figure 6). An Arduino Uno R3 microcontroller digitized the analog signals from the custom circuit at 10 kHz. The microcontroller then rectified the signals before applying a moving average with a window set to 50 data points. Windows larger than 50 data points resulted in sEMG signals that did not indicate muscle activation, whereas smaller windows would result in a noisier signal. Thus, the window of 50 data points was a compromise between noise and the sensitivity of the sEMG DAQ to muscle activation. In order to ensure compatibility with various PC platforms across different studies, UnoJoy! a firmware and software package developed for the Arduino Uno, mapped the averaged sEMG signals to a universal serial bus (USB) joystick control scheme so that an amplitude of 0 mV was assigned to the centered joystick position, while a peak amplitude was assigned to an extreme rightward joystick position [81]. Since the steering of the driving simulator (DBS2™, Bohemia Interactive) was controlled by keyboard commands, the laptop (Panasonic CF-LX6 laptop, 14 inch 1920 × 1080 resolution screen) executed JoyToKey software to convert maximum rightward joystick input into the keyboard command to steer rightward at a set SWR [82]. Whenever a test subject is connected to the DAQ, the game controller calibration software in Windows 10 was executed so that the center joystick position and maximum rightward joystick position could be set. Then, in order to mitigate sEMG signal interference and to calibrate for the maximum sEMG amplitude of a given test subject, the threshold for sEMG control signals was set in JoyToKey from 10% to 30% of the peak joystick input signal, i.e., the maximum peak average rectified sEMG signal resulting from isometric contraction lasting up to 1 s. For some test subjects, it was less likely for the JoyToKey software to recognize sEMG input at thresholds above 30%, and thus the threshold was not increased beyond this percentage during training or experimental trials.

**Figure 7:**
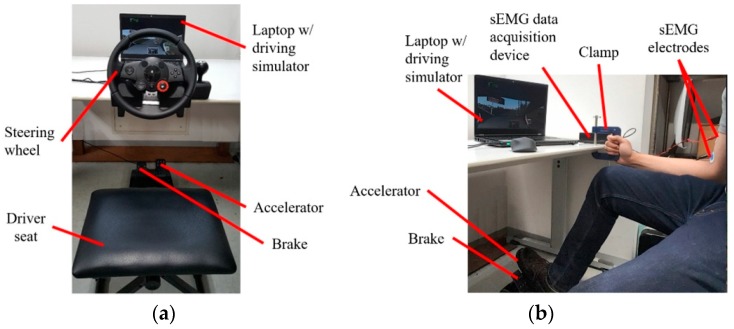
Driving simulator setups for (**a**) game steering wheel and (**b**) steering assistance interface.

The experimental setup for the force feedback game steering wheel (Driving Force™ GT) consists of: a laptop that runs the driving simulator, the steering wheel assembly, and the brake and accelerator pedal assembly (Figure 7a). In the case of the steering assistance interface, braking and acceleration were controlled by the same pedal assembly, but the steering wheel assembly was replaced by the clamp and sEMG equipment (Figure 7b, Videos 1c and 1d).

### 2.4. Driving Scenarios

**Figure 8:**
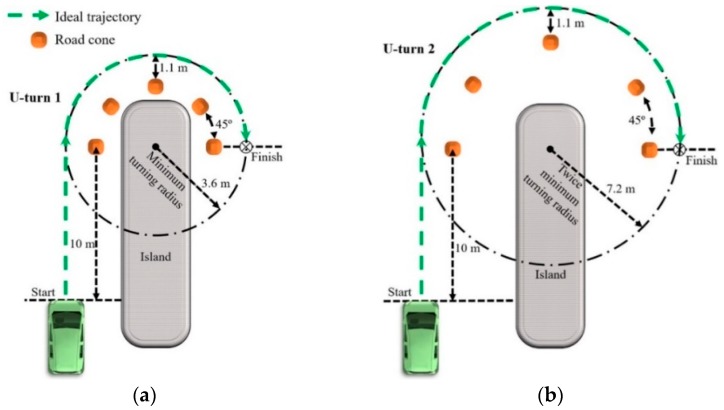
Driving simulator scenarios consisting of U-turns with radii of curvature equal to (**a**) 3.6 m and (**b**) 7.2 m. Note: figures not to scale.

The game steering wheel and steering assistance interface were used to perform two types of U-turns. U-turn 1 had a radius of curvature of 3.6 m, which is equal to the minimum turning radius of the simulated automobile (Figure 8a), whereas the radius of U-turn 2 was twice as long (Figure 8b). Supplementary Video S1a–d demonstrate the completion of the simulated U-turns. For each U-turn, participants were instructed to accelerate the simulated car from a full stop at the starting line. A right turn was initiated through an isometric contraction of the right arm biceps brachii or by rotating the game steering wheel rightward with both hands, in accordance with the majority of studies associating rapid steering wheel rotation with high shoulder joint forces [4,83]. Rotation of the game steering wheel in the opposite direction of the turn, or a subsequent isometric contraction, caused the simulated car to exit the turn. The brake pedal was applied after the simulated car crossed the finish line. 

The change in SWA from 0° to a maximum of 65° throughout U-turn 1 (Figure 8a) is associated with high shoulder joint forces when holding a steering wheel [4]. Given that the steering assistance interface was developed to enable turns without holding the steering wheel, U-turn 1 was completed with steering assistance to test the path following accuracy as shoulder joint forces resulting from manual steering wheel rotation are avoided. Furthermore, since the turn is performed at the maximum SWA, reduced steering portability at high SWAs would be also be avoided, if the interface were used in an actual car.

Turning the game steering wheel to the maximum SWA to execute U-turn 1 (Figure 8a) precludes steering correction involving rightward steering wheel rotation. In contrast, U-turn 2 (Figure 8b) requires the car to steer at twice the minimum turning radius so that the SWA is below the maximum SWA. Thus, steering correction may be performed by turning the steering wheel rightward when the car understeers, i.e., steers wider than the ideal trajectory. U-turn 2 was therefore designed to test path following accuracy in a scenario that allows for the correction of understeer. 

### 2.5. Lateral Vehicle Dynamics

Oversteer and understeer are vehicle characteristics that occur during steady-state cornering along a circular path [6,34]. After a vehicle undergoes a transient phase to transition from a longitudinal trajectory into a circular path, the lateral position of the vehicle enters steady-state and remains constant. Understeer or oversteer occur when the lateral position of the vehicle changes along the circular path. On the other hand, if steady-state cornering is maintained at the lowest possible speed along the circular path, the front road wheels are at the Ackermann steer angle for that path. For example, if the simulated vehicle follows the ideal trajectory of U-turn 1 with a constant lateral position and the lowest constant speed, the front road wheels are at the Ackermann steer angle for U-turn 1 (Figure 8a). 

“Ideal trajectory” refers to the path that is followed by an ideal vehicle that can instantaneously transition from a longitudinal trajectory to a steady-state circular trajectory. However, since automobiles always require time to transition, a transient phase necessarily occurs before reaching steady-state. A longer transient phase extends the time to reach steady-state, resulting in higher lateral error. In order to minimize the transient phase, the steering wheel could be rotated by the driver as quickly as possible. In the case of the steering assistance interface, the shortest possible transient phase available in the driving simulator is 0.1 s. Since the maximum SWA of the simulated vehicle is 65° and the SWR of 720 deg/s is attainable through commercially available steering actuators, dividing the maximum SWA by the SWR of the actuator yields an approximate transient phase of 0.1 s [38]. Thus, the minimum transient phase of the driving simulator was set to 0.1 s as a feasible value. 

In contrast to U-turn 1, a smaller steady-state SWA applies to U-turn 2 (Figure 8). Since the steering ratio of the driving simulator is 1:1, the SWA is equal to the front steer angle, δ_F_, of the front wheels. Based on the radius of curvature, R, of U-turn 2 and the wheelbase length, *l*, the front steer angle could be approximated as follows [6,84]:

δ_F_ = *l*/R (1)

Since R is 7.2 m and *l* is 4.1 m, dividing *l* by R and converting to degrees yields an approximate value of 33° for δ_F_ and the SWA. Thus, in order to perform U-turn 2, the interface is programmed so that +δ_Hset_ is 33° to the right for the driving simulator. 

Although it is possible to correct for understeer and oversteer in the driving simulator through longitudinal deceleration or acceleration, respectively, there is also an option to adjust the SWA in the case of the game steering wheel. Driving simulator trials with U-turn 2 (Figure 8b) are used to determine if the adjustment of the SWA with the game steering wheel, as oppose to no SWA adjustment with the sEMG interface, would increase path following accuracy. One type of trial is completed by operating the game steering wheel to perform U-turn 2, whereas the other type of trial would consist of the completion of U-turn 2 with the sEMG interface in place of the game steering wheel. If the game steering wheel is significantly more accurate than the sEMG interface, as a result of SWA adjustment, then the ability of manual SWA adjustment to increase accuracy would be confirmed. On the other hand, if the game steering wheel has comparable or significantly less accuracy than the sEMG interface, then this ability is not confirmed. 

If the transient phase of the sEMG interface were different than that of the game steering wheel, any difference in accuracy between the two interfaces in the case of U-turn 2 (Figure 8b) could be attributed to a set of conditions that includes the difference in transient phases and the availability of manual SWA correction with the game steering wheel. Since the experimental trials with U-turn 2 are only intended to assess the effect of manual SWA correction on path-following accuracy, the effect of different transient phases must be minimized. Therefore, the transient phase of the sEMG interface was adjusted to be similar in duration to that of the game steering wheel. A survey of data collected from driving simulator trials and field testing indicates that the average time to perform a steering correction ranges from 0.38 s to 0.57 s with an average of approximately 0.5 s [85]. Hence, the transient phase for the sEMG interface was set to 0.5 s to approximate the transient phase associated with manual steering wheel control. Data from driving simulator trials would determine if the difference between the approximated and actual transient phases produces a significant difference in path following accuracy between the two interfaces. 

### 2.6. Experimental Trials

In order to facilitate experimental replicability, driving simulator trials were completed by 16 nondisabled human participants with intact limbs, since amputated limbs have scar tissue or other confounding variables that may affect sEMG measurement and, consequently, experimental results [77]. All test subjects provided their informed written consent before participating in the trials. The study was completed in accordance with the Declaration of Helsinki and was approved by the Ethics Committee of the Interfaculty Initiative in Information Studies, Graduate School of Interdisciplinary Information Studies, The University of Tokyo (No. 14 in 2017). 

Four of the test subjects had prior driving simulator experience, while all test subjects had an average of 0.7 standard deviation (SD) 0.9 years of actual driving experience. All participants had standard automobile driver’s licenses issued by the Japanese government. The participants were between 19 and 24 years of age, with an average age of 21 years. 

All participants were right-handed, except for two people. Given that previous research has found no significant intra-subject difference in sEMG between the biceps brachii muscles of the dominant and non-dominant arms during isometric contraction, it was expected that handedness of the participants would not significantly influence experimental results [86]. 

Although one out of the 16 test subjects was female, and the sEMG amplitude of the biceps brachii has been previously observed during isometric contraction to be significantly lower in females than in males, the sEMG input threshold of the steering assistance interface was calibrated for each participant to mitigate the effect of inter-subject amplitude variability on the ability of the interface to detect input (Section 2.3) [87]. 

U-turns illustrated in Figure 8 were performed by the participants to test the following hypotheses:

1. For the U-turn with a radius of curvature equal to 3.6 m, the steering assistance interface is comparable or greater in path-following accuracy to the game steering wheel.
2. For the U-turn with a radius of curvature equal to 7.2 m, the steering assistance interface is comparable or greater in path following accuracy to the game steering wheel, even if the sEMG interface is used without steering wheel correction.

As indicated in Table 1, four experimental conditions were performed to test the hypotheses. Data from conditions 1 and 2 were used to test the first hypothesis, whereas data from conditions 3 and 4 test the second hypothesis. Each of the 16 participants were trained to complete all conditions. Training began with a slide presentation containing written instructions on the performance of each U-turn in Figure 8 and videos of an expert user demonstrating the operation of game steering wheel, pedal assembly, and the steering assistance interface (Videos 1a to 1c). Then the participants practiced with the driving simulator by successfully completing each U-turn twice with each interface. After training was finished, the participants completed the experimental conditions. The participants were divided into groups of 4 so that a 4 × 4 balanced Latin square could be used to randomize the order in which each participant completed the conditions [88]. Each participant attempted each condition five times. An attempt was successful if the simulated car passed the first and last road cones without running over the island. In order to prevent brake pedal depression from interrupting the constant speed of steady-state cornering, drivers were instructed to refrain from pressing the brake pedal during the turn. If correction for oversteer or understeer was necessary, only the game steering wheel, if available, or the accelerator could be used. 

Trajectories from the first three successful attempts of each condition were used for data analysis to ensure that enough data would be available for processing. Median trajectories across trials were plotted on two-dimensional coordinate planes to compare the trajectories of the interfaces with respect to the ideal trajectories. Note that 1.1 m is the shortest distance between the edge of any road cone and the ideal trajectories (Figure 8). The lateral error of the simulated automobile was determined by the absolute value of the difference between 1.1 m and the shortest distance between the actual trajectory and the edge of a given road cone. The lateral error was found for each of the five road cones in each scenario. For each condition in Table 1, the intertrial median lateral error across all participants was calculated as the path following accuracy. The interquartile range (IQR) was calculated as the extent to which data are spread about the intertrial median lateral error [89].

Comparisons between interfaces were made with statistical analyses conducted in MATLAB. The Shapiro–Wilk test was performed, where *p* < 0.05, i.e., the hypothesis that the population of the data is normally distributed is rejected, if *p* is less than 0.05. Since *p* < 0.05 for the data from conditions 1 to 4 in Table 1, the data were determined to not be normally distributed [90]. Hence, the non-parametric Wilcoxon signed-rank test was selected to determine whether there is any statistical difference in path-following accuracy between the game steering and the sEMG interface. The significance level for the Wilcoxon signed-rank test was *p* < 0.05, i.e., if *p* < 0.05 for one of the tested U-turn types, there is a significant difference between the two interfaces, and one rejects the hypothesis that the median intertrial difference in lateral error is zero between the interfaces [91]. For hypothesis (1), data from conditions (1) and (2), were analyzed to determine if there was a statistically significant difference in path following accuracy between the sEMG interface and the game steering wheel. As for hypothesis (2), data from conditions (3) and (4) were analyzed to determine if there was a significant difference in path following accuracy between the two interfaces. 

## 3. Results

**Figure 9:**
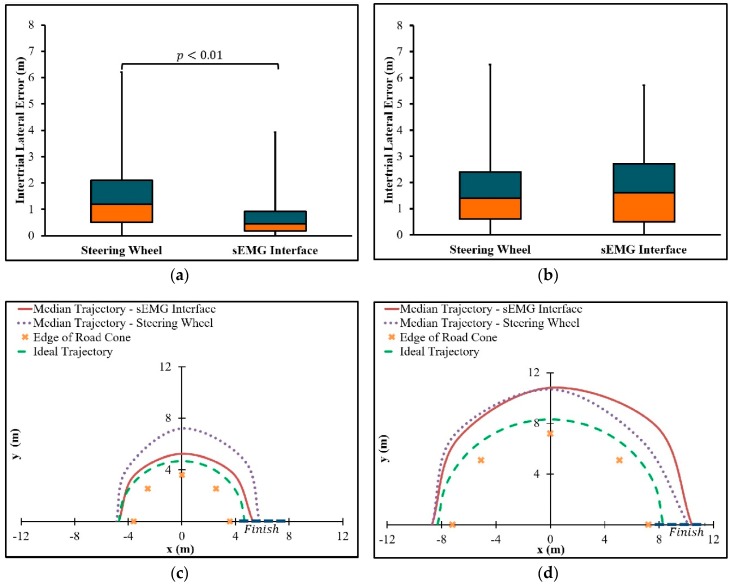
Path-following accuracy of sEMG interface and game steering wheel for U-turns with radii of curvature equal to (**a**) 3.6 m and (**b**) 7.2 m. Corresponding steering trajectories are shown for U-turns with radii of curvature equal to (**c**) 3.6 m and (**d**) 7.2 m.

Based on intertrial lateral error data across all participants, the sEMG interface was compared to the game steering wheel with respect to path following accuracy (Figure 9). For U-turn 1 (Figure 8a), with a radius of curvature equal to 3.6 m, the sEMG interface with the SWA, +δ_Hset_, set to 65° was more accurate than the game steering wheel. The difference between the sEMG interface and the game steering wheel, with respect to the intertrial median lateral error, was statistically significant with *p* < 0.01. In the case of U-turn 2 (Figure 8b), with a radius of curvature equal to 7.2 m, the sEMG interface, with +δ_Hset_ equal to 33°, was comparable in accuracy to the game steering wheel, since there was no statistically significant difference in path following accuracy. 

Median trajectories for each interface are shown in Figure 9. Throughout the U-turn with a radius of curvature of 3.6 m, the median trajectory of the sEMG interface is closer to the ideal trajectory than the median trajectory of the game steering wheel (Figure 9c). During the first half of the U-turn with a radius of curvature equal to 7.2 m, the sEMG interface has a median trajectory that tends to be closest to the ideal trajectory, whereas the median trajectory of the game steering wheel tends to be closest in the second half of the turn (Figure 9d).

Based on calculations from experimental data, intertrial median errors and IQRs are summarized in Table 2. For the U-turn with a radius of curvature equal to 3.6 m (Figure 8a), the sEMG interface had a lower intertrial median error and IQR than the game steering wheel. However, for a radius of curvature equal to 7.2 m (Figure 8b), the sEMG interface had a higher intertrial median error and IQR than the game steering wheel. 

## 4. Discussion

According to the intertrial median lateral errors in Figure 9, the sEMG interface is at least comparable to the game steering wheel for all the tested U-turns. Therefore, hypotheses (1) and (2) are confirmed (Section 2.6). The median trajectories in Figure 9 support potential explanations for the confirmed hypotheses. In the case of the U-turn where the radius of curvature is 3.6 m, the median trajectory of the sEMG interface is closest to the ideal trajectory (Figure 9c). Since the game steering wheel must be held at the maximum SWA throughout the turn in order to follow the ideal trajectory, it is not expected that any understeer was corrected by steering further rightward. The inability to correct for understeer during the turn could explain why the trajectory of the simulated automobile appears to deviate away from the ideal trajectory during the turn. Notice that the trajectory of the sEMG interface exhibits less deviation than the game steering wheel with respect to the ideal trajectory. This lower deviation may result from a briefer transient phase. Recall that the transient phase of the sEMG interface was set to 0.1 s, in contrast to the previously measured transient phase of 0.268 SD 0.065 s for steering wheel interfaces [4]. As expected from the discussion on transient phases in Section 2.5, the sEMG interface is associated with steady-state cornering that occurs sooner during the turn than for the game steering wheel, thus resulting in a more accurate trajectory. 

The transient phase of the sEMG interface in the case of U-turn 2 (Figure 8b) was set to approximate the transient phase of the game steering wheel. As expected, the interfaces have similar trajectories in the first half of the turn (Figure 9d). This result is consistent with the transient phase of sEMG interface being set to 0.5 s to approximate the transient phase of the game steering wheel (Section 2.5). Since the setting of 0.5 s is an estimate based on empirical data from driving simulations and actual vehicles, the transient phases of the game steering wheel and sEMG interface coincide with some actual automobiles. 

Unlike U-turn 1 (Figure 8a), understeer correction with the game steering wheel is allowed by U-turn 2 (Figure 8b). Another possible source of correction is the increase of vehicle speed by pressing the accelerator. This correction may have resulted in the decreased lateral error of the game steering wheel trajectory in the second half of the turn. Conversely, the lack of understeer correction with either the accelerator or the steering wheel may account for the higher lateral error of the sEMG interface at the same part of the turn. Nevertheless, there is no statistically significant difference in intertrial median lateral error between the two interfaces. 

The trajectory data from the driving simulator trials suggest that steering assistance could provide path-following accuracy that is comparable to a steering wheel interface, even in scenarios where steering wheel correction may be performed. Accuracy in the context of the tested scenarios (Figure 8) is dependent on steady-state cornering. Although there may be undesired forces within actual steering systems, i.e., steering compliances, that may hinder steady-state cornering by causing deviation from a circular path, modern steering systems can be improved through design optimization in order to mitigate the effects of steering compliances [6,92]. Therefore, the simulated automobile represents actual automobiles with fully functioning steering systems that have undergone design optimization. 

Since data were collected from 16 participants with Japanese driver’s licenses, future studies could recruit a larger sample of test subjects to more accurately represent the population of drivers in Japan. Participants in this study were all persons without disabilities, and thus the results are at least relevant to drivers with one healthy arm. It is expected that a driver who has hemiplegia or upper limb amputation affecting only the left side of the body could use an unaffected right upper limb to operate the sEMG interface with accuracy that is comparable to current experimental results. As for sEMG input from upper limbs with unilateral amputations above the right wrist or right elbow, comparable operation is possible, if the residual biceps brachii repeatedly provides sEMG signals that are similar in amplitude and activation period to the sEMG signals of intact limbs. Otherwise, future studies including drivers with amputations could be compared to this study to quantify any statistically significant difference in path-following accuracy. As the interface continues to be implemented for drivers with disability, the suitability of the interface in relation to specific drivers and automobiles would be assessed by specialists of disability, such as occupational therapists and physiatrists [93]. 

Based on present and future evaluations of the sEMG interface with respect to path following accuracy, further interface modifications could be realized. One planned modification is the replacement of the disposable wet electrodes with reusable dry electrodes for more efficient mounting and to eliminate the need for drivers to remove conductive gel and electrode adhesive from the skin after using the interface. Additions to the interface handle would include a photoelectric motion sensor to detect the hand of the driver, as explained in Section 2.1, and a vibrotactile device to provide feedback on the SWA of the steering wheel (Figure 2d). 

## 5. Conclusions

A steering assistance interface with sEMG electrodes underwent driving simulator trials to evaluate path-following accuracy relative to a game steering wheel. Although further testing was required to confirm the accuracy of the sEMG interface in most cases, trajectory data from the trials indicated that the steering assistance interface was at least comparable to the game steering wheel, with respect to the tested U-turns (Figure 9). For a U-turn that allows for understeer correction, the steering assistance interface provided comparable accuracy, even in the absence of steering wheel correction. The current study demonstrates how sEMG sensors could be used to address the risk of shoulder overload and reduced steering portability without significantly compromising path-following accuracy. Future research could utilize the findings of this study to realize accurate sEMG controlled automobile steering for persons with disability.

## Figures and Tables

**Table 1:** Experimental conditions for driving simulator.

Conditions	Interface Type	Radius of Curvature of U-Turn (m)
1	sEMG	3.6
2	Game steering wheel	3.6
3	sEMG	7.2
4	Game steering wheel	7.2

**Table 2:** Summary of results from driving simulator trials.

Conditions	Interface Type	Radius of Curvature of U-Turn (m)	Intertrial Median Error (m)	Interquartile Range (IQR) (m)
1	sEMG	3.6	0.5	0.8
2	Game steering wheel	3.6	1.2	1.6
3	sEMG	7.2	1.6	2.2
4	Game steering wheel	7.2	1.4	1.8

## Supplementary Materials

The following are available online at https://www.mdpi.com/1424-8220/19/6/1308/s1.
